# Spontaneous Neck Hematoma in a Patient with Fibromuscular Dysplasia: A Case Report and a Review of the Literature

**DOI:** 10.1155/2013/352830

**Published:** 2013-09-26

**Authors:** Oded Cohen, Moshe Yehuda, Meital Adi, Yonatan Lahav, Doron Halperin

**Affiliations:** ^1^Department of Otolaryngology, Kaplan Medical Center, Rehovot, Israel; ^2^Department of Radiology, Kaplan Medical Center, Rehovot, Israel

## Abstract

*Background*. Fibromuscular dysplasia (FMD) is a vascular disease that may present as aneurysms in the cervical arteries. Spontaneous neck hematoma is a rare life threatening medical condition. This is the first report of neck hematoma in a patient with FMD. *Methods and Results*. We present a case of a 69-year-old woman, with diagnosed cervical FMD and a 3-day history of sore throat and neck pain, who presented with enlarging neck hematoma. No active bleeding was noticed on CT angiography, airway was not compromised, and patient was managed conservatively. Next day, invasive angiography was performed, and no bleeding vessel was demonstrated. Patient has improved and was discharged after 5 days of hospitalization. We have discussed the different etiology of this condition, focusing on systemic vascular diseases. *Conclusion*. Complaint of neck pain in a patient with a FMD should raise suspicion for possible neck hematoma. Conversely, spontaneous neck hematoma without clear etiology should raise suspicion for a systemic vascular disease.

## 1. Introduction

Spontaneous neck hematoma is a rare medical condition which may be life threatening [1–5]. The literature regarding the subject is based solely on case reports. The classic symptoms of cervical hematoma are tracheal and esophageal compression, tracheal displacement, and subsequent appearance of subcutaneous bruising or swelling in the neck, known as the Capps triad [1, 2, 6]. Other symptoms such as dysphagia, hoarseness, and pain have also been described [1, 7].

The etiology of nontraumatic spontaneous neck hematoma includes rupture of aneurysms [1, 8, 9], rupture of parathyroid adenoma [3, 4, 7], infection [10], and an underlying coagulopathy [11].

Fibromuscular dysplasia (FMD) is a noninflammatory, nonatherosclerotic vascular disease that involves small and medium-sized arteries [12, 13]. Its pathogenesis is idiopathic and involves true proliferation of smooth muscle cells and fibrous tissue [14]. The diagnosis of FMD is established by histopathology or angiography [12–15]. The classic angiographic pattern is that of irregular caliber with alternating segments of narrowing and dilatation, also known as the “strings of beads” sign, which has been observed in over 80% of the cases [12, 14, 15].

FMD most commonly involves the renal (60%) and cervicocephalic arteries (30%) [13, 14]. The latter could result in an ischemic or hemorrhagic stroke and cervical artery dissection and could be associated with intracerebral aneurysms [12, 14, 15]. The prevalence of symptomatic cervicocephalic FMD is estimated to be 0.002% [14]. In most series of patients with cervical FMD, the mean age at diagnosis was over 50 years [12, 15].

Approximately 95% of cervical FMD involves the internal carotid artery (ICA), often bilaterally, classically affecting the middle and distal portions of the ICA [12, 15].

We present a case of spontaneous cervical hemorrhage in a 69-year-old woman previously diagnosed with cervico-cephalic FMD. To our knowledge, this is the first report of spontaneous neck hemorrhage due to a vascular systemic disease and might also be the first description of cervical hemorrhage as a potential complication of FMD [12–15].

## 2. Case Report

The patient, a 69-year-old Caucasian woman, was admitted to our department for a spreading hematoma in her neck. She complained of throat and neck pain as well as progressive dysphagia which had started 3 days prior to her admission. She has denied dyspnea or shortness of breath. She has reported that a day earlier a small hematoma appeared in her neck, which has rapidly spread. She had sought medical consultation on the day that her throat pain began and had received oral antibiotics.

Her medical history were significant for two ICA aneurysms which have been diagnosed in 2002 following a head CT that has been performed due to headaches. Following the scan, the patient underwent a cerebral angiography and coiling of an aneurysm located in the paraophthalmic segment of the right ICA (see Figure 2). A 6 × 7 mm aneurysm in her left ICA was managed conservatively. Typical FMD changes have been noted in the left ICA. In 2009, following headache complaints, the patient returned for medical followup, and an increased diameter of the left ICA aneurysm (up to 12 mm) was diagnosed on MRA, with dissecting features. The patient underwent stent insertion in the left ICA. Follow-up imaging revealed normal blood flow in both ICAs with no new aneurysms diagnosed.

The patient's medications included 100 mg aspirin and 100 mg amiodarone per day prescribed for atrial fibrillation.

On admission, the patient had normal vital signs and was afebrile. The oral cavity and oropharynx were normal in appearance. There was a large subcutaneous hematoma extending from the thyroid gland and to the right third rib inferiorly. Marked sensitivity was noted while palpating the right lateral neck, superior to the cricoid cartilage. A slight tracheal deviation to the left has been noted. The patient did not demonstrate any signs of airway compromise such as stridor or dyspnea. Fiber-optic laryngoscopy demonstrated normal glottis and clear pyriform sinuses, without signs of hematoma or laryngeal compression.

Standard laboratory tests, including a coagulation profile, were within the normal range. Blood calcium level has been obtained in order to rule out a parathyroid adenoma and was 8.2 mg/dL (normal range 8.4–10.2).

Urgent neck ultrasound (US) has been performed, revealing a large hematoma displacing the right lobe of the thyroid anteriorly.

**Figure 1:**
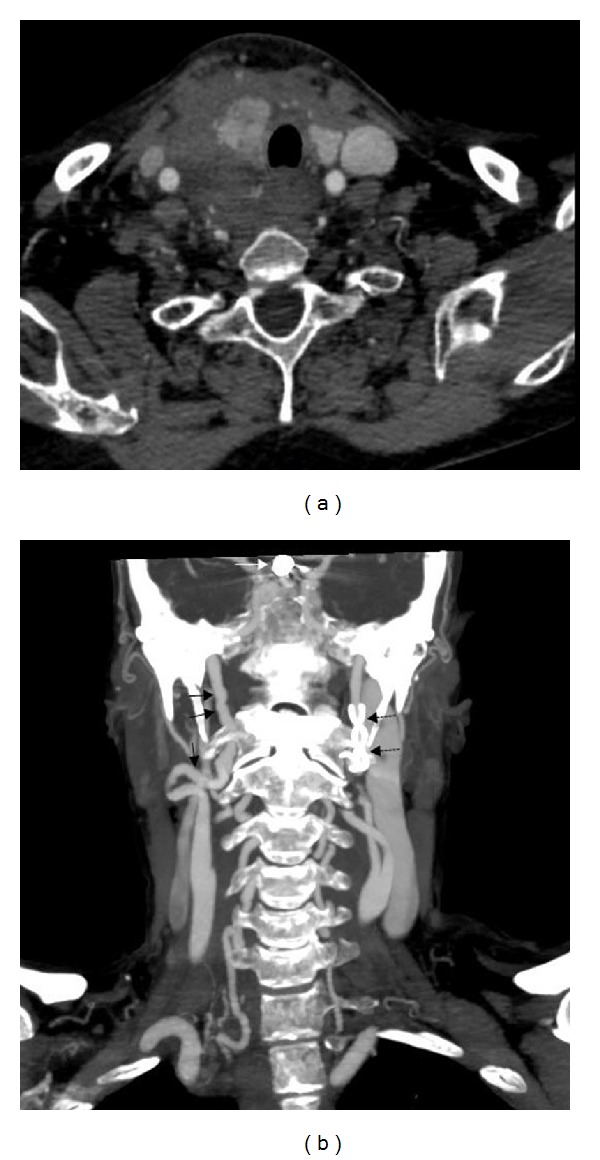
Contrast-enhanced angiographic CT (CTA). (a) Contrast-enhanced CT angiography in axial view shows the large right cervical hematoma causing anterior displacement of the right thyroid lobe and slight tracheal deviation to the left. (b) CT angiography in coronal maximal intensity projection (MIP) reformation showing the classical “strings of beads” sign in the right ICA (black arrows), typical for FMD, the stent in the left ICA (black dashed arrows), and the coil in the paraophthalmic segment of the right ICA (black arrow). No active bleeding is seen.

**Figure 2:**
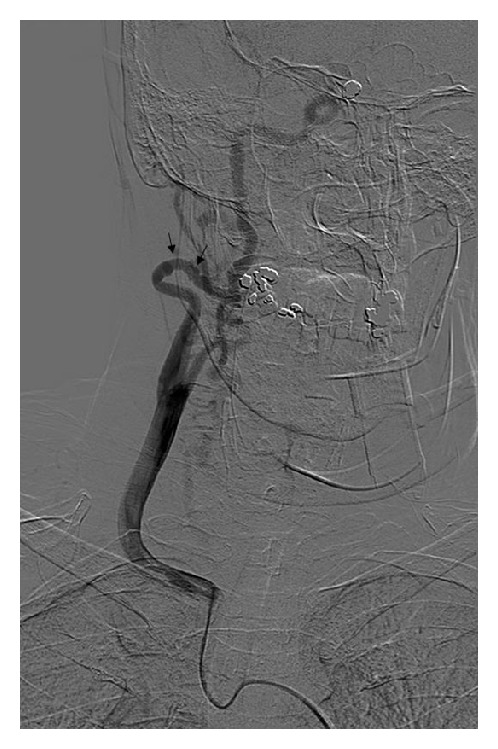
Angiography of the cervical arteries. Conventional angiography demonstrates typical “strings of beads” in the right ICA (black arrows) without active bleeding.

After consulting with vascular surgeons, a contrast CT angiography was performed (Figure 1), demonstrating a large right neck hematoma that has caused anterior displacement of the right thyroid lobe and tracheal deviation to the left. No active bleeding was demonstrated. The aortic arch and the right and left ICA were intact.

Based on the stable clinical presentation and vital signs, lack of any sign of possible airway compromise, and lack of active bleeding on imaging, a decision to manage the patient conservatively was made. IV antibiotics were administrated as prophylaxis. During the night, a progression in the hematoma has been noticed, yet vital signs have remained stable throughout the night, with no complaints regarding airway distress or neck pain.

**Figure 3:**
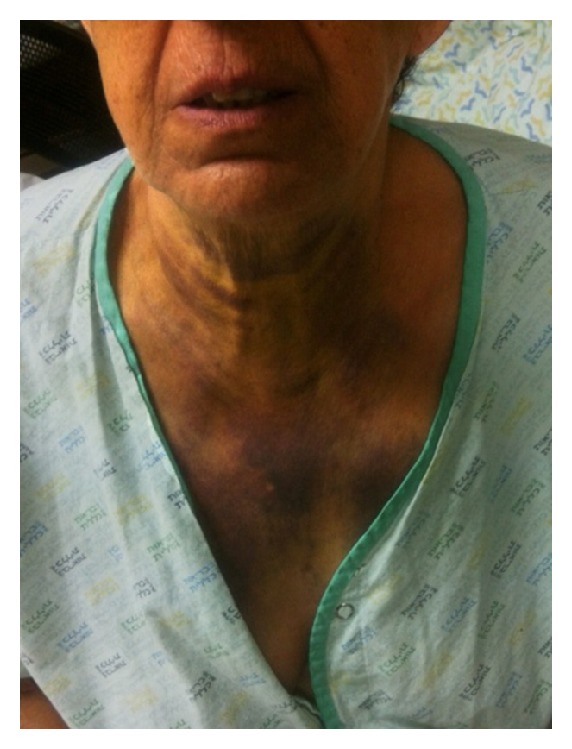
The picture was taken on the second day of hospitalization: hematoma had spread to the upper chest reaching the right breast.

Next morning, hoarseness has been noted, as well as an expansion of the hematoma to midchest and to the right areola (Figure 3). Fiber-optic laryngoscopy revealed small hematomas on the right true and false vocal cord, as well as on the right arytenoid. A bed side US demonstrated heterogenic area without clear borders between the thyroid gland and the hematoma. In contrast to the signs mentioned above, the patient remained without any respiratory distress and without even a slight sign of airway compromise. Nevertheless, the patient was transferred to the intensive care unit (ICU), and conventional angiography was performed (Figure 1) in an attempt to localize and control the source of the bleeding.

Angiography revealed normal arteries of the right cervico-cephalo region: both the internal and the external carotid were intact as well as the aorta and the left common carotid. The lack of active bleeding in the major arteries has raised a dilemma whether to attempt entry into the small branches of the right external carotid. Since FMD is known to increase the risks of dissection, following an invasive vascular procedure [14], the small arteries have not been visualized.

The patient remained in a stable condition in the ICU and later returned to the ENT department. She was discharged on the fifth day of hospitalization, with stable signs and marked improvement of hoarseness, swelling, and size of the hematoma.

Additional clinical improvement was noted 10 days after her discharge, by the attending doctor in the outpatient clinic.

## 3. Discussion

The patient has presented a typical progression of neck hematoma: at first, pain was the main complaint, for which medical attention was sought, and antibiotic treatment was given. Early neck hematoma can be misdiagnosed as viral pharyngitis due to sore throat without shortness of breath [16]. As the hematoma progressed, the patient has been admitted for further investigation and treatment. Despite several urgent imaging studies, no bleeding vessel was found. At this point, the question of preventive intubation and secure airway became the main dilemma in the management of the patient. Unlike Stenner et al. [1], who found that cervical hemorrhage should optimally be managed by intubation, diagnosis, and therapy in this following order, we prioritize clinical condition assessment, similar to Chin et al. [2]. During her hospitalization, the patient never demonstrated clear evidence of airway compromise, such as posterior wall bulging [17], and intubation was not indicated.

The majority of spontaneous neck hematomas are caused by ruptured aneurysms [1, 6, 8, 9]. Silent lesions in apparently unaffected arteries are a common finding in FMD [18], and rupture of microaneurysm in patients with FMD is a documented phenomenon found in autopsy of the basilar artery [19]. Moreover, ruptured aneurysms are not necessarily seen during angiography [1]. Therefore, although reports are scarce [8], we believe that it is reasonable to assume that the hemorrhage has been a result of a ruptured microaneurysm in one of the thyroid vessels, in the inferior thyroid artery, being the most probable one [1].

Various presentations have been related to cervico-cephalic FMD in large reviews [12, 14], such as retinal or cerebral ischemic events, intracranial aneurysms, subarachnoid hemorrhage, cervical or intracranial dissections, amaurosis fugax, Horner's syndrome, and cranial-nerve palsies. Nonspecific symptoms include headache, tinnitus, vertigo, lightheadedness, and syncope [12]. In our literature review, we did not find any mention of spontaneous neck hematoma as a possible presentation or complication of cervico-cephalic FMD.

Other systemic vascular and connective tissue diseases, such as polyarteritis nodosa (PAN), Ehlers-Danlos syndrome (EDS) type IV, and Marfan's syndrome, have been reported to have similar visceral aneurysms [20–23] and are considered in the differential diagnosis of FMD [12, 13]. After reviewing the literature for possible similar reports in these diseases, we have found only a single report of a 28-year-old woman with EDS type IV who has developed an anterior neck hematoma caused by spontaneous rupture of the external carotid artery [24].

## 4. Conclusion

Spontaneous hemorrhages secondary to a systemic vascular disease are extremely rare. To the best of our knowledge, this is the first report of a neck hematoma presenting as a potential complication of FMD. We urge physicians to maintain a high index of suspicion when a patient with FMD presents with a cervical complaint for the possibility of a hematoma in progress. In patients with a spontaneous neck hematoma, without clear etiology, the diagnosis of a systemic vascular disease including FMD should be considered.
